# Association of SARS-CoV-2 infection with long-lasting increase in circulating IL-32 levels

**DOI:** 10.3389/fimmu.2026.1739258

**Published:** 2026-02-06

**Authors:** Lorenzo Miano, Elena Sinopoli, Alessandro Cherubini, Chiara Suffritti, Serena Pelusi, Fatima Rahmeh, Giuseppe Enzo Lamorte, Flora Peyvandi, Francesco Blasi, Giacomo Grasselli, Alessandra Bandera, Roberta Gualtierotti, Daniele Prati, Luca Vittorio Carlo Valenti

**Affiliations:** 1 Università degli Studi di Milano, Department of Pathophysiology and Transplantation, Milan, Italy; 2 Precision Medicine and Biological Resource Center, Fondazione IRCCS Ca’ Granda Ospedale Maggiore Policlinico Milano, Milan, Italy; 3 SC Medicine – Haemostasis and Thrombosis, Fondazione IRCCS Ca’ Granda Ospedale Maggiore Policlinico Milano, Milan, Italy; 4 Pulmonology and Cystic Fibrosis, Fondazione IRCCS Ca’ Granda Ospedale Maggiore Policlinico Milano, Milan, Italy; 5 Anesthesiology and Intensive Care Unit, Fondazione IRCCS Ca’ Granda Ospedale Maggiore Policlinico Milano, Milan, Italy; 6 Infectious Diseases, Fondazione IRCCS Ca’ Granda Ospedale Maggiore Policlinico Milano, Milan, Italy

**Keywords:** COVID-19, cytokine storm, IL-32, inflammation, long-covid

## Abstract

**Background & aims:**

Severe Acute Respiratory Syndrome Coronavirus 2 (SARS-CoV-2) infection has a wide spectrum of clinical presentations ranging from asymptomatic viral replication to hyper-inflammatory syndrome and respiratory failure and can trigger immune disorders and long-COVID. Interleukin-32 (IL-32) is a pro-inflammatory cytokine induced during viral infections and chronic pulmonary disease.

**Aim:**

Aim of this study was to investigate the impact of the SARS-CoV-2 pandemic and severe COVID-19 on circulating IL-32 levels.

**Study design:**

Observational retrospective biomarker study.

**Patients & methods:**

We analyzed 949 healthy blood donors (pre-pandemic and pandemic-era) and 212 patients hospitalized due to severe COVID-19 during the first five infection waves. IL-32 levels were measured by ELISA.

**Results:**

Pandemic-era blood plasma donors showed a +0.78 ± 0.09 log_10_ pg/ml mean increase in IL-32 (pandemic-era 2.91 ± 0.05 vs. pre-pandemic 2.14 ± 0.07 log_10_ pg/ml, p<0.0001). COVID-19 patients exhibited a similar elevated IL-32 compared to unexposed controls (+0.29 ± 0.11 log_10_ pg/ml, p=0.016; 2.43 ± 0.08 hospital admission vs. pre-pandemic). Among patients, mean IL-32 was higher in first-wave patients (2.68 ± 0.11 log_10_ pg/ml) than later waves (2.12 ± 0.11 log_10_ pg/ml). In setting of severe COVID-19, IL-32 levels were associated with corticosteroids administration (estimate1.99 ± 0.50; p<0.0001), whereas decreased during the later waves of infection (-0.56 ± 0.16; p=0.0005) and with age (estimate -0.01 ± 0.01; p=0.020). No links were found with sex, Intensive care unit admission, comorbidities, or mortality. A subset of the COVID patient cohort was tested for pro-inflammatory biomarkers: IL-32 displayed an inverse correlation with patients’ neutrophil-to-lymphocyte ratio (NLR) (estimate -0.23 ± 0.81; p=0.005) and not with IL-6 and biomarkers of endothelial dysfunction (n=42, p=NS). In patients with available follow-up (n=96), IL-32 remained stable up to one-year post-discharge (+0.03 ± 0.12 log_10_ pg/ml, p=0.970; 2.55 ± 0.15 hospital admission vs. follow-up 3–12 months 2.58 ± 0.15 log_10_ pg/ml).

**Conclusions:**

IL-32 levels increased following COVID-19, especially during the initial severe wave, and correlated with some markers of inflammation. IL-32 remained elevated up to one-year post-discharge, suggesting ongoing inflammation and supporting its potential as a biomarker for long-term sequelae.

## 1 Introduction

Severe Acute Respiratory Syndrome Coronavirus 2 (SARS-CoV-2) is a virus belonging to the family Coronaviridae, causing a severe form of viral pneumonia that was reported for the first time in December 2019 (1, 2). COVID-19 was defined as the disease caused by SARS-CoV-2 by the World Health Organization (WHO) on February 11th 2020, and on March 11th 2020, it was declared a pandemic (3, 4). SARS-CoV-2 infection can lead to a respiratory syndrome associated with pneumonia, presenting with a range of clinical phenotypes from mild to critical (5, 6). Severe cases are marked by acute respiratory distress, often necessitating hospitalization and mechanical ventilation (7).

The pathophysiology of severe COVID-19 is characterized by the abnormal release of multiple proinflammatory cytokines (cytokine storm) that contribute to alveolar exudation and lung tissue damage (8, 9). Indeed, overexpression of the proinflammatory cytokines IL-1, IL-6, IL-8 and TNF-α and low expression of IFN-γ have been found in severe COVID-19 patients (10–13).

Interleukin-32 (IL-32) has been recognized as a proinflammatory cytokine that plays a role in the body’s antiviral response to viral infections. The IL32 gene is located at chromosome 16 and the transcript undergoes alternative splicing, resulting in six variants: IL-32α, IL-32β, IL-32γ, IL-32δ, IL-32ϵ and IL-32ζ (14). IL-32γ, the most proinflammatory among isoforms, is involved in host immune response to monocyte cell differentiation (15). IL-32 can stimulate various immune cells by activating signaling pathways such as nuclear factor kappa B (NF-κB) and mitogen-activated protein kinases (MAPKs). It is produced by epithelial cells as well as several immune cells, including natural killer (NK) cells, T cells, and monocytes. Although the exact mechanisms underlying IL-32 signaling are not yet fully understood, it is known to promote the production of inflammatory cytokines and chemokines—such as IL-6, TNF-α, IL-8, and MIP-2/CXCL2—by immune cells (16).

Augmented circulating IL-32 was observed in patients with Influenzavirus A (IAV) (17) and Hepatitis B virus (HBV) infection (18), as well as in Human Papillomavirus (HPV)-positive cervical cancer cells (19). Furthermore, a strong correlation between IL-32 and chronic obstructive pulmonary disease (COPD) has been reported, with IL-32 increased in plasma, bronchial lavage fluid (BAL), and induced sputum of COPD patients compared to healthy individuals (20). Recent studies detected independently an analogous and distinct increment of IL-32 levels in patients following COVID-19 infection, compared to groups of healthy controls (21–24).

IL-32 activity has also been proven to be implicated in metabolic diseases. IL-32 overexpression has been detected in people with metabolic dysfunction associated steatotic liver disease (MASLD) presence and severity (25), and correlated with liver disease pathogenesis (26).

Moreover, IL-32 has been detected in endothelial cells (EC) (27) where it was involved in endothelial development and remodeling. Foremost, IL-32 promotes angiogenesis, supporting new blood vessel formation, and it also modulates the expression of adhesion molecules like ICAM-1 and cytokines such as IL-1α, IL-6, and IL-8, which are essential for endothelial activation and inflammation (28). In keeping, circulating IL-32 levels have been linked to impaired blood pressure regulation in individuals with MASLD (29).

Nonetheless, the evidence regarding the role of IL-32 in the cytokine storm after infection remains sparse and fragmented, particularly with respect to its circulating levels. Furthermore, the mechanistic link between elevated IL-32 concentrations during cytokine storms and metabolic disorders has yet to be fully elucidated.

With this in mind, the aim of this study was to compare circulating IL-32 levels between patients hospitalized for severe COVID-19 at the peak of infection compared to healthy blood donors before and after pandemic, and to examine the association with clinical features and the evolution during follow-up.

## 2 Patients and methods

### 2.1 Study cohorts

The reference cohort was made up of 949 apparently healthy individuals, presenting for blood donation from June 2019 to February 2021 at the Transfusion Medicine unit of the Fondazione IRCCS Ca’ Granda Hospital (Liver-Bible-Cohort 2021). The Liver-Bible-Cohort 2021 has previously been described (29) and the clinical features of this cohort are summarized in **Table 1**.

**Table 1:** Liver-bible-cohort 2021.

Baseline characteristics	Pre COVID	Post COVID	p
n	314	635	
Sex, M	270 (86.0)	521 (82.0)	0.150
Age, years	53.3 (6.5)	54.2 (6.3)	0.035
BMI, units	28.7 (2.9)	28.4 (2.9)	0.111
Platalets, 10^3^/μL	238.4 (48.7)	233.2 (53.0)	0.144
Ferritin, ng/mL	96.3 (90.1)	119.6 (120.9)	0.003
CRP, mg/dL	0.3 (0.3)	0.2 (0.4)	0.358
Hypertension, yes	210 (66.9)	444 (69.9)	0.380
Anti-hypertensive therapy, yes	122 (38.9)	132 (20.8)	<0.001
Type 2 diabetes, yes	12 (3.8)	23 (3.6)	1.000

Data are shown as n (%) and Mean ± SD, as appropriate. Summary statistics of healthy blood donors, enrolled from June 2019 to July 2021. 30^th^ January 2020, the first COVID-19 patient registered in Italy, was used as the threshold date to distinguish blood donors screened before (Pre COVID) and after (Post COVID) COVID-19 exposure. Clinical features recorded from subjects: platelets, ferritin, C-reactive protein (CRP), body mass index (BMI), hypertension, anti-hypertensive treatment, type 2 diabetes. p, p-value.

Since the first COVID-19 cases were officially registered in Italy on January 30, 2020, we used this date to separate blood donors who donated before (pre-pandemic) and throughout (pandemic-era) the pandemic. The absence of active infections at the time of sample collection was confirmed in all Liver-Bible-Cohort 2021 subjects by serological screening.

**Figure 1:**
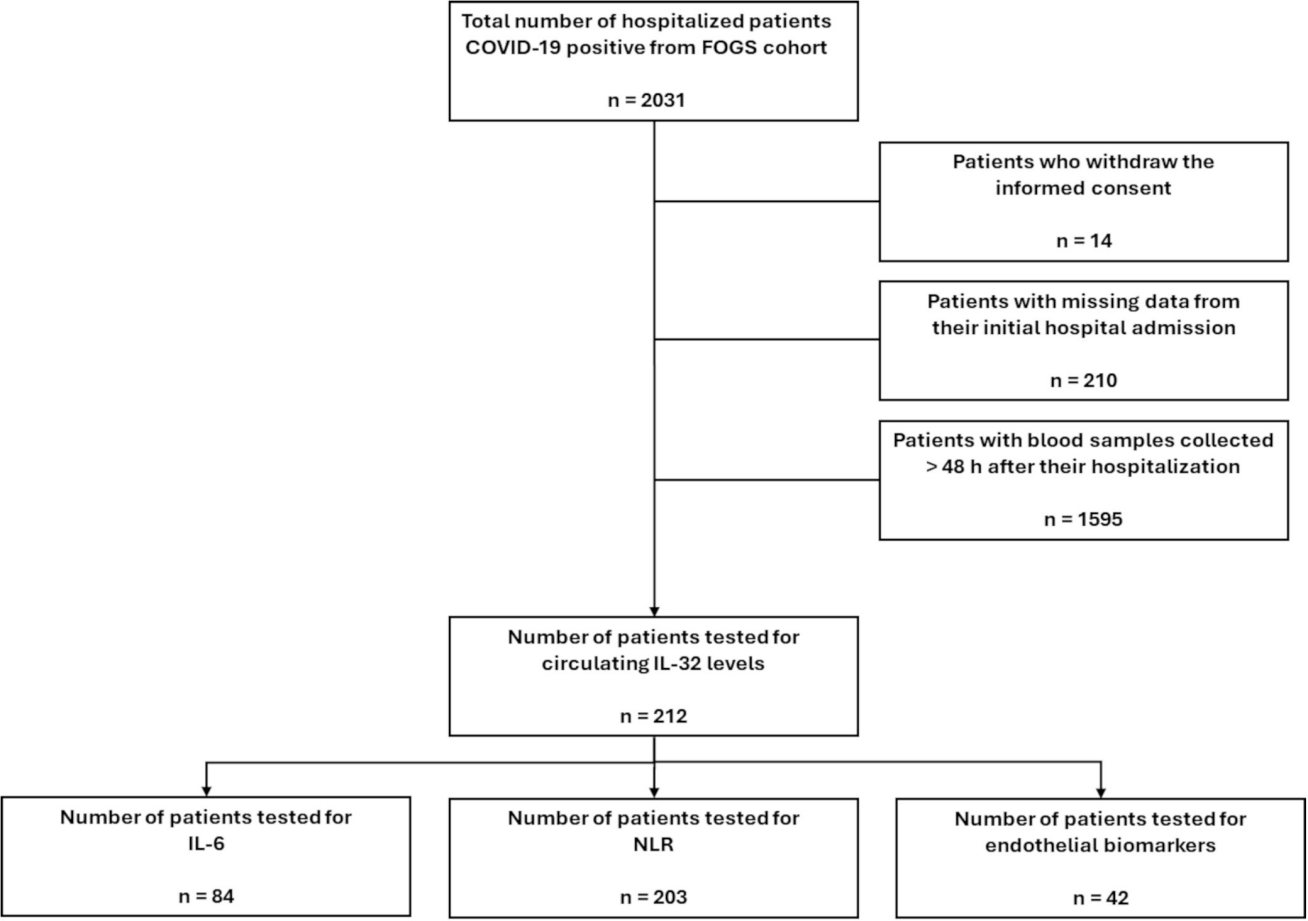
Flow diagram of hospitalized COVID-19 patients enrollment from the FOGS cohort, following the study inclusion criteria.

The retrospective cohort of hospitalized COVID-19 patients (Hospitalized COVID Patients, **Table 1**) has been determined following the STROBE guidelines for observational cohorts. The cohort was selected starting from the Fondazione Genomic Study (FOGS) collection of data and biological samples of hospitalized people included in COVID-19 registries at the Fondazione IRCCS Ca’ Granda Hospital. We considered as main criteria for the enrolment in the study patients aged over 18 years, admitted between March 2020 and February 2022 to the Infectious Disease Unit and the Intensive Care Unit (ICU) (Registro COVID, nCoV-ICU), with a confirmed COVID-19 diagnosis (positive RT-PCR for SARS-CoV-2 and/or positive nasopharyngeal swab and/or positive BAS/BAL). Only patients who provided informed consent were included. Patients for whom data were not collected at least 48h after their hospitalization, or whose data were missing due to initial admission to other facilities or units, were excluded from the study. In conclusion, a total of 212 patients met these inclusion criteria for the study (**Figure 1**).

The study was approved by the Ethics Committee of Milano Area 2, Fondazione IRCCS Cà Granda of Milan (approval number 342_2020). All participants enrolled in the study provided written informed consent prior to participation.

Given the relevance of cardiometabolic comorbidities known to influence circulating IL-32 levels, we collected data on arterial hypertension and related antihypertensive treatments, corticosteroid therapies, diabetes, and previous episodes or known pre-existing cardiovascular diseases. Furthermore, we classified COVID-19 disease severity based on ICU admission status.

The neutrophil-to-lymphocyte ratio (NLR) was calculated as the ratio between absolute neutrophil and lymphocyte counts obtained from the complete blood cell count.

Interleukin-6 (IL-6) levels were quantitatively measured using Elecsys IL-6 test, following the manufacturer’s instructions.

Whenever possible, the plasma samples collected for each patient were clustered in two groups: acquired at 24 and 48 hours after hospitalization (Acute phase) and acquired during visits at 3, 6, and 12 months post-discharge (Follow-Up phase). Infection waves were delineated in accordance with the criteria established by Protezione Civile Italiana (30). For the purposes of this study, emphasis was placed on stratifying data from the initial wave (March 2020 to September 2020) in comparison to the other four subsequent waves.

A complete overview of the clinical features of the Hospitalized COVID Patients cohort is reported in **Table 2**, while **Supplementary Table S1** describes follow-up patients for the same clinical characteristics, stratified for the first wave and subsequent waves.

**Table 2:** Hospitalized COVID patients.

Baseline characteristics	Discharged	Deceased	p	Overall
n	137	75		212
Sex, M	90 (65.7)	48 (64.0)	0.923	138 (65.1)
Age, years	61.2 (13.7)	77.1 (10.9)	<0.001	66.8 (14.9)
ICU, yes	21 (15.3)	2 (2.7)	0.009	23 (10.8)
Corticosteroid treatment, yes	2 (2.5)	4(5.3)	0.245	6 (0.05)
Hypertension, yes	70 (75.3)	61 (83.6)	0.268	131 (61.8)
Anti-hypertensive therapy, yes	61 (62.9)	47 (67.1)	0.686	108 (50.9)
Cardiovascular diseases, yes	15 (16.7)	27 (38.6)	0.003	42 (19.8)
Type 2 diabetes, yes	18 (20.0)	20 (28.6)	0.282	38 (17.9)
Wave, first	99 (72.3)	26 (34.7)	<0.001	125 (59.0)

Data are shown as n (%) and Mean ± SD, as appropriate. Summary statistics of hospitalized COVID-19 patients, enrolled from March 2020 to February 2022. The cohort was stratified according to patients’ survivability, considering the following clinical variables: Sex, Age, the severity of the disease that led to Intensive Care Unit access (ICU), and if patients were hospitalized during the period between March 2020 to September 2020 or later (Wave). Clinical features recorded from subjects: corticosteroid treatment, arterial hypertension, anti-hypertensive treatment, cardiovascular diseases, and type 2 diabetes. p, p-value.

From this cohort, a subgroup of 42 patients was also included in a parallel study investigating endothelial biomarkers.

### 2.2 Measurement of circulating IL-32

For plasma collection, peripheral blood collected in EDTA tubes was centrifuged at 2000 x g for 15 min and immediately stored at -80°C at the Fondazione Biological Resource Center (POLI-MI Biobank, which is part of the Italian node of Biobanking and Biomolecular Resources Research Infrastructure, BBMRI). Plasma samples were thawed, maintaining the cold chain to obtain aliquots. IL-32 plasma levels were measured using Human IL-32 DuoSet ELISA kit (Cat. N° DY3040, R & D Systems, Minneapolis, MN, USA), following manufacturer’s instruction. The assay is designed to detect IL-32α, IL-32β, and IL-32γ with a detection range of 78.5–5000 pg/mL. Kit storage followed the manufacturer’s instructions. Briefly, Samples were diluted 1:2-1:128 in cold PBS and measured in duplicate. Analyses were repeated, increasing dilution ratios when IL-32 values outside the standard curve were detected. Optical density was measured at 450 nm and 540 nm (as reported in the manufacturer’s instructions) using TECAN Infinite F200 PRO instrument (Männedorf, Switzerland). The minimal detectable concentration was 39 pg/mL. We employed the same methodology and procedures for data normalization and variance analysis of raw ELISA measurements as outlined in prior publications (29). Additionally, in instances where IL-32 concentrations were undetectable, a default value of 10 pg/mL was assigned.

### 2.3 Measurement of endothelial biomarkers

Soluble thrombomodulin (TM) was measured using a sandwich ELISA kit (Human Thrombomodulin/BDCA-3 Quantikine ELISA Kit, Cat. N° DTHBD0, R&D Systems). Vascular endothelial growth factor (VEGF) in plasma was assessed by means of the Human VEGF Quantikine ELISA Kit (Cat. N° DVE00, R&D Systems). Vascular cell adhesion molecule-1 (VCAM-1) was measured in plasma using the Human VCAM-1/CD106 Quantikine ELISA Kit (Cat. N° DVC00, R&D Systems). Endoglin was assessed by means of the Human Endoglin/CD105 Quantikine ELISA Kit (Cat. N° DNGD00, R&D Systems). All measurements have been performed following manufacturer’s instruction. Optical density was measured using a VersaMax microplate reader (Molecular Devices, San Jose, CA).

### 2.4 Statistical analysis

Since IL-32 values were not normally distributed, log_10_-transformation was applied for normalization.

Analogously, IL-6 and NLR values were normalized by applying the Rint transformation.

Comparisons of continuous variables between independent groups (Liver-BIBLE pre-pandemic, Liver-BIBLE pandemic-era, and Hospitalized COVID cohort) were performed using the unpaired Wilcoxon test. For continuous variables within paired groups of patients, where samples were available both at hospitalization during the Acute phase and at the Follow-Up phase, the paired Wilcoxon test was used.

The Liver Bible Cohort 2021 served as the reference population to calculate the minimum sample size needed to detect significant changes of one log_10_ unit in distribution comparisons using the Wilcoxon test (power=0.8, two-tailed alpha=0.05). A sample size of at least 114 subjects was determined to achieve the desired statistical power, consisting of 38 controls and 76 patients. Categorical subsets and proportional fractions for each tested group were defined by low or high levels of circulating IL-32, with high levels corresponding to values above the third quartile of the total IL-32 distribution (>3.237 log10 pg/ml).

Possible correlations between variables (sex [M, F], age, COVID-19 severity [ICU, Not ICU], corticosteroid treatment [yes, no], anti-hypertensive therapies [yes, no], wave of infection [first, second-fifth], previous pathologies [type 2 diabetes, or arterial hypertension, or cardiovascular diseases], survivability [outcome, dead], pro-inflammatory biomarkers [IL-6, NLR], endothelial biomarkers (TM, VEGF, VCAM-1, endoglin]) and IL-32 levels were evaluated performing generalized linear model (GLM). P-values less than 0.05 were considered significant. R (v. 4.2.2) was used to perform all the statistical analyses.

## 3 Results

### 3.1 Change in IL-32 levels in apparently healthy individuals after the COVID-19 pandemics

**Figure 2:**
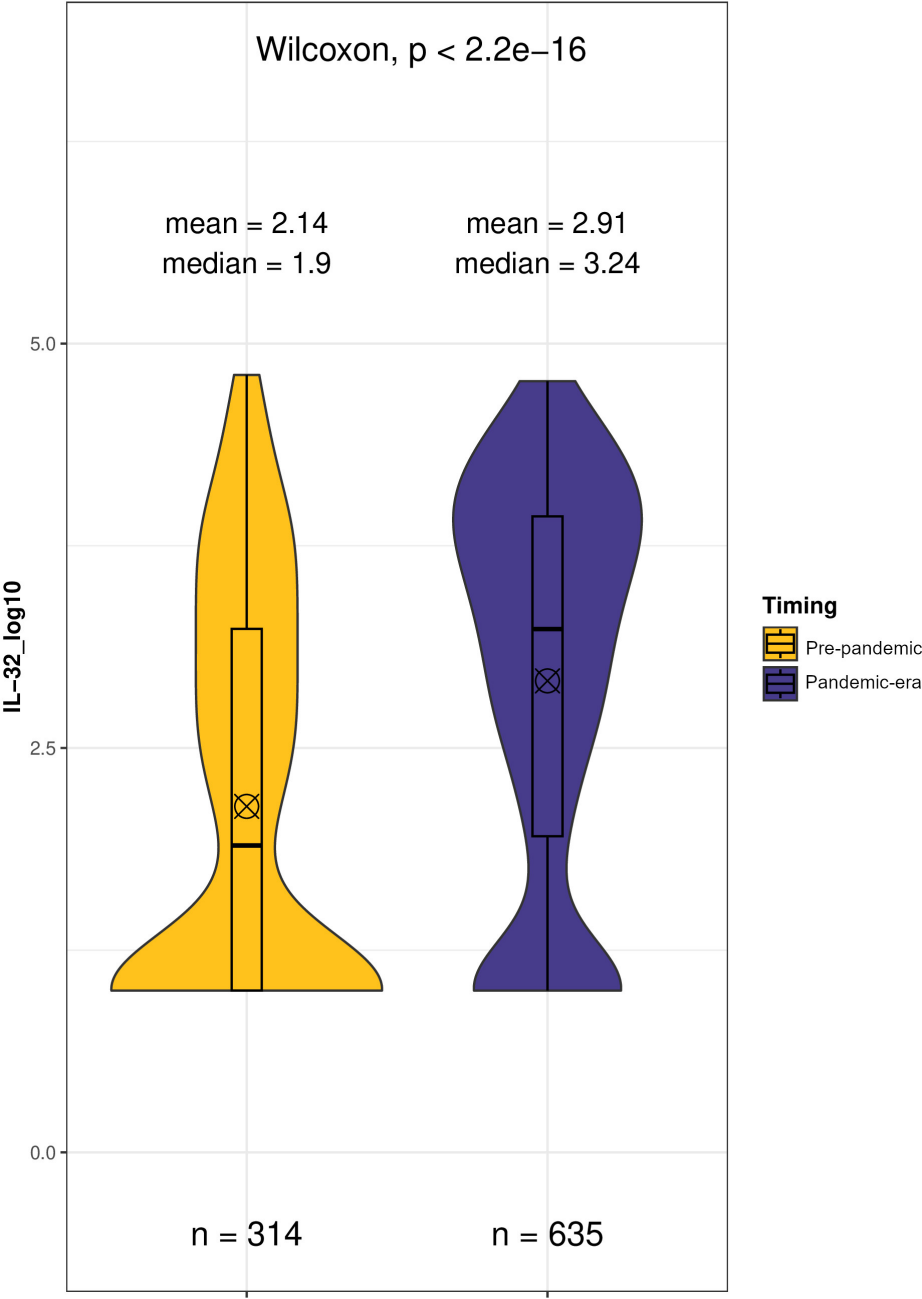
Comparison of IL-32 level distributions in 949 healthy blood donors screened before (Pre-pandemic) and after (Pandemic-era) 30th January 2020, the first COVID-19 cases were officially registered in Italy. Wilcoxon test of log10-transformed IL-32 concentrations (pg/mL). ⊗ Mean;Median.

To test whether SARS-CoV-2 infection may affect circulating IL-32 levels irrespective of disease course, we first analysed cytokine levels in apparently healthy blood donors sampled before and after the onset of the first wave in Italy. Results are shown in **Figure 2**. Comparison of pre-pandemic and pandemic-era groups in Liver-BIBLE showed a significant general increase in circulating IL-32 in blood donors after January 2020 (Δ Mean ± SE: +0.78 ± 0.09 log_10_ pg/ml, p=2.2x10-16; pre-pandemic: 2.14 ± 0.07 log_10_ pg/ml vs. pandemic-era: 2.91 ± 0.05 log_10_ pg/ml). These data suggest that the spread of infection was associated with subclinical inflammation and an increase in IL-32 even in apparently healthy individuals in the population.

### 3.2 Clinical features of the study cohort and circulating IL-32 distribution

The Hospitalized COVID-19 patients’ cohort had ultimately enrolled 212 patients, primarily over 60 years old (66.8 ± 14.9), with the majority being male (138 individuals, 65.1%) (**Table 2**). Among them, 23 patients (10.8%) were admitted to the ICU due to COVID-19 severity or complications. A total of 125 (59.0%) patients were hospitalized during the first period of the pandemic (FIRST wave). The most prevalent comorbidity was hypertension, reported in 131 patients (61.8%), of whom 108 were receiving antihypertensive treatment. Additionally, 6 patients (0.1%) were on corticosteroid therapy. Seventy-five patients (35.4%) died from COVID-19 during hospitalization, while follow-up was available for 96/137 of surviving patients (70.1%).

**Figure 3:**
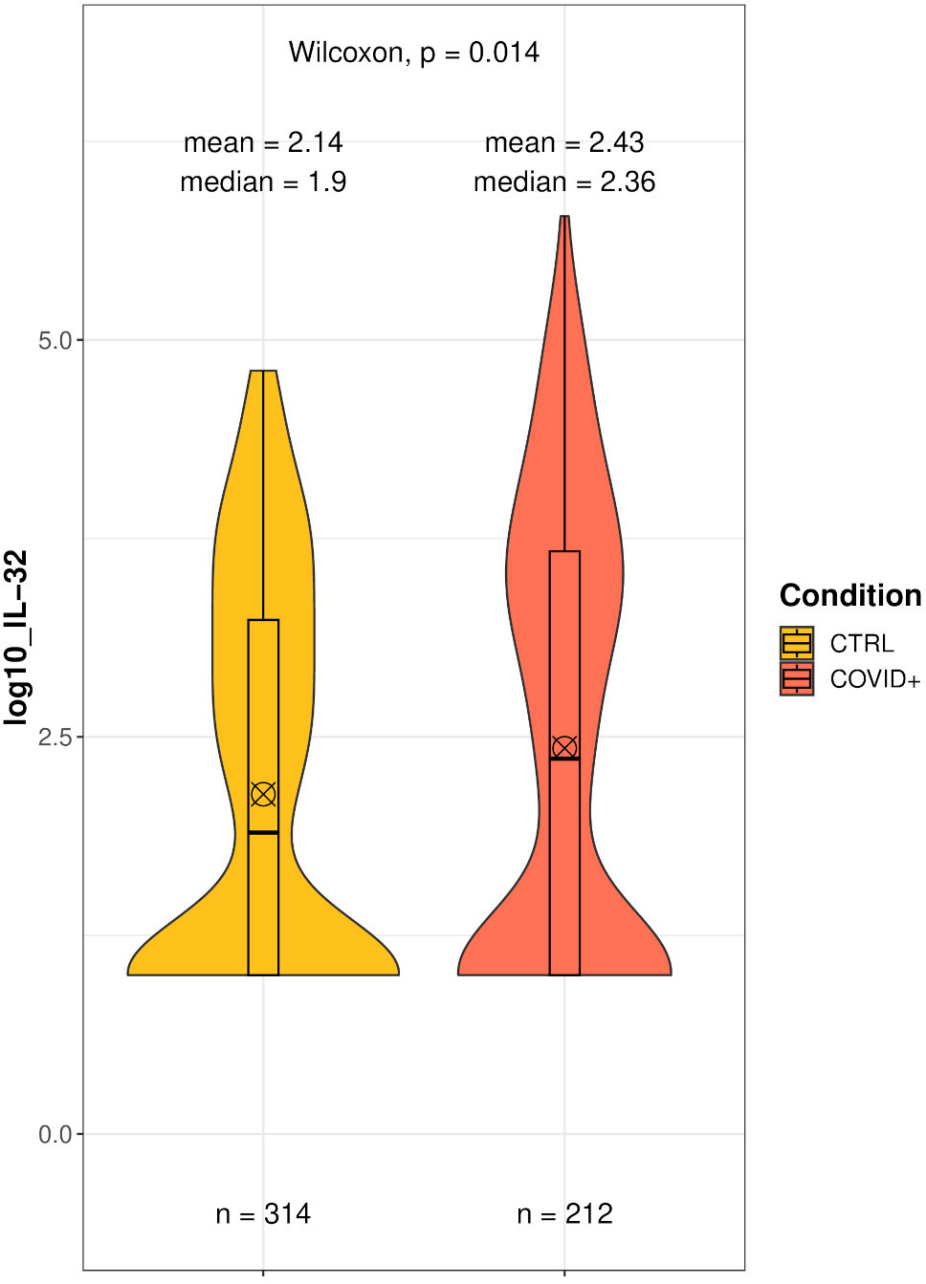
Comparison of IL-32 level distributions between 314 healthy blood donors, unexposed to SARS-CoV-2 (CTRL), and 212 patients due to COVID-19 during their hospitalization (COVID+). Wilcoxon test of log10-transformed IL-32 concentrations (pg/mL). ⊗ Mean;Median.

**Figure 3** shows the distribution of circulating IL-32 in hospitalized patients with COVID-19 (2.43 ± 0.08 log_10_ pg/ml). Consistent with previous data, we detected a highly variable range in IL-32 measurements, which spanned over almost 5 log_10_ pg/ml (Maximum value: 5.78 log_10_ pg/ml).

When we compared IL-32 levels to those of healthy blood donors before the pandemic, we detected significantly higher levels of IL-32 in patients compared to controls (+0.29 ± 0.11 log_10_ pg/ml, p=0.016), supporting the notion that IL-32 levels increase following SARS-CoV-2 exposure (**Figure 3**).

### 3.3 Circulating IL-32 levels are associated with the use of corticosteroids, age, and wave of infection among hospitalized patients

Next, we examined the clinical correlates of circulating IL-32 in hospitalized COVID-19 patients, first using univariate linear models to test associations.

The strongest positive association was observed between IL-32 levels and the severity of inflammation, as detected by the clinical indication to start corticosteroid treatment (estimate 1.99 ± 0.50; p=7.71×10^−5^, **Table 3**). Conversely, IL-32 levels were significantly lower in COVID-19 patients infected during the subsequent waves compared to those infected during the first wave (estimate -0.56 ± 0.16; p=0.0005).

**Table 3:** Correlation of circulating IL-32 with clinical variables.

Univariate				Multivariate (Age+Corticosteroids+Wave)		
Baseline characteristics	Estimate	SE	p	Estimate	SE	p
Sex, M	-0.28	0.17	0.104			
Age, years	-0.01	0.01	0.020	-0.01	0.01	0.074
ICU, yes	-0.25	0.21	0.247			
Cardiovascular diseases, yes	-0.01	0.21	0.943			
Hypertension, yes	-0.16	0.22	0.485			
Anti-hypertensive therapy, yes	-0.15	0.19	0.424			
Corticosteroid treatment, yes	1.99	0.50	<0.0001	1.86	0.49	0.00016
Type 2 diabetes, yes	-0.25	0.22	0.274			
Outcome, deceased	-0.24	0.17	0.147			
Wave, first	-0.56	0.16	0.0005	-0.54	0.16	0.001

Univariate Generalized Linear Models (GLMs) for the log_10_ of the amount of circulating IL-32 (pg/mL), considering the following covariates: Sex, Age, ICU admission, and wave of infection. Multivariate GLM for the log_10_ of the amount of circulating IL-32 (pg/mL) adjusted for the following covariates: Age, and if patients were hospitalized during the beginning of the pandemic (Wave). p, p-value; SE, standard error.

Additionally, advancing age was modestly associated with a decline in circulating IL-32 (estimate -0.01 ± 0.01; p=0.020).

Multivariable regression analyses (accounting for corticosteroid treatment, infection wave, age, **Table 3**) corroborated the independent positive correlation between IL-32 levels and corticosteroid treatment (estimate 1.86 ± 0.49; p=0.0002), as well as the inverse association with hospitalization during the first wave of infection relative to subsequent waves (estimate -0.54 ± 0.16; p=0.001). Although the negative correlation between age and IL-32 levels persisted, it did not reach statistical significance (estimate = -0.01 ± 0.01; p=0.074).

When considering IL-32 concentration as the dependent variable in COVID patients, no statistically significant associations were observed with the other covariates characterizing the patient cohort: sex, disease severity (ICU), hypertension, cardiovascular comorbidities, or mortality.

### 3.4 Inflammatory response and endothelial biomarkers

To explore the potential involvement of IL-32 in the inflammatory response, we initially evaluated its associations with two key inflammatory markers—IL-6 (n=84) and the neutrophil-to-lymphocyte ratio (NLR) (n=203)—in participants from the Hospitalized COVID Patients cohort for whom complete data were available (**Supplementary Table S2**). This approach allowed us to assess both cytokine-based (IL-6) and cellular (NLR) indicators of inflammation in relation to circulating IL-32 levels. Correlation analyses indicated no significant relationship between IL-32 and IL-6 (estimate -0.17 ± 0.13; p=0.21). In contrast, circulating IL-32 was significantly and inversely associated with NLR (estimate -0.23 ± 0.81; p=0.005).

Given that IL-32 has also been implicated in angiogenesis and endothelial biology, we further expanded our analysis to explore the potential relationship between circulating IL-32 concentrations and markers of endothelial activation and injury. For this purpose, we focused on a subgroup of 42 patients from the same Hospitalized COVID Patients cohort (**Supplementary Table S2**). In total, 72 plasma samples were collected from these patients at multiple time points throughout hospitalization and follow-up, allowing for a dynamic assessment of biomarker changes over time. We then performed correlation analyses between IL-32 and a panel of endothelial biomarkers, including thrombomodulin (TM), vascular endothelial growth factor (VEGF), vascular cell adhesion molecule-1 (VCAM-1), and endoglin. None of these markers exhibited a statistically significant correlation with IL-32 levels (**Table 4**).

**Table 4:** Correlation of circulating IL-32 with endothelial biomarkers.

Baseline characteristics	n	Univariate		
		Estimate	SE	p
IL-6	84	-0.17	0.13	0.213
NLR	203	-0.23	0.81	0.005
TM	42	-0.12	0.14	0.394
VEGF	42	0.13	0.14	0.365
VCAM-1	42	-0.08	0.14	0.584
Endoglin	42	0.14	0.14	0.315

Univariate Generalized Linear Models (GLMs) of the log10 of the amount of circulating IL-32 (pg/mL) for different pro-inflammatory biomarkers: Interlukyn-6 (IL-6) and neutrophil-to-lymphocyte ratio (NLR), and for different endothelial biomarkers: thrombomodulin (TM), vascular endothelial growth factor (VEGF), vascular cell adhesion molecule-1 (VCAM-1), and Endoglin. n, number of tested subjects; p, p-value; SE, standard error.

### 3.5 IL-32 remains elevated in COVID-19 patients at one year follow-up

**Figure 4:**
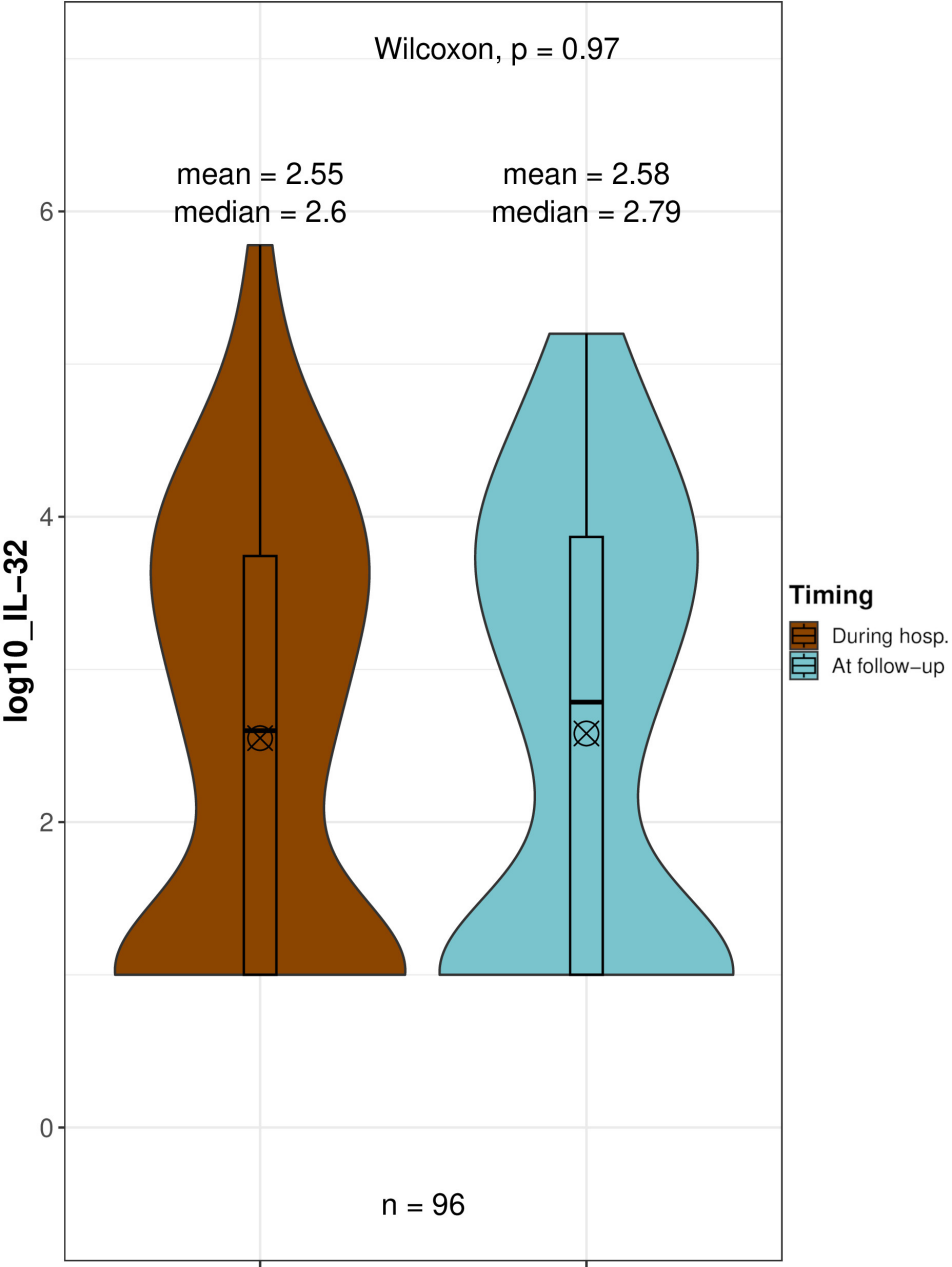
Comparison of IL-32 level distributions in 96 patients due to COVID-19 during their hospitalization and at follow-up visits (3, 6, and/or 12 months post-discharge). Paired Wilcoxon test of log10-transformed IL-32 concentrations (pg/mL). ⊗ Mean;Median.

To investigate the longitudinal trend of IL-32 levels in COVID-19 patients, we analyzed those with follow-up visits by comparing their IL-32 measurements taken at hospitalization with levels measured post-discharge. IL-32 levels remained stable over time in these patients (+0.03 ± 0.12 log_10_ pg/ml, **Figure 4**), with no statistically significant change observed (p = 0.970).

This outcome suggests that elevated circulating IL-32 can persist for several months after recovery from COVID-19.

## 4 Discussion

In this study, we examined the impact of COVID-19 on the circulating levels of IL-32, an atypical proinflammatory cytokine (31). Beyond its role in viral infections, including COVID-19, IL-32 contributes to immune and cardiometabolic disorders (25, 32), and it is also involved in promoting angiogenesis (28) and regulating endothelial functions (27). Since recent data pointed to persistent elevation of IL-32 in the lung following severe COVID-19 (33), we hypothesized that IL-32 might be involved in the pathogenesis of autoimmune disorders (34) and persistent symptoms following COVID-19, namely long-COVID (34, 35), irrespective of the severity of the acute infection.

First, we showed that circulating IL-32 increased in apparently healthy individuals with metabolic dysfunction presenting for blood donation following the emergence of COVID-19 in Italy. This enabled us, in preliminary analysis, to establish a reference baseline for IL-32 levels in apparently healthy individuals who were certainly unexposed to SARS-CoV-2 (**Figure 2**, pre-pandemic subjects). Subsequently, comparison of these pre-pandemic healthy donors with individuals diagnosed with COVID-19 during the first waves of infection, before the advent of vaccination programs and of direct anti-viral therapies, revealed an increase in IL-32 levels among COVID-19 patients. Although SARS-CoV-2 serological data at the time of sample collection were unavailable for the study dataset, our previous analyses in the same population reported a statistically significant increase in anti-SARS-CoV-2 seroconversion rate in a comparable blood donor cohort with similar demographics and sampling timeframe (36). Indirectly, this finding aligns with observations in our pandemic-era subgroup, suggesting parallel elevations in both anti-SARS-CoV-2 antibodies and IL-32 levels. These findings support the notion that IL-32 levels rise as consequence of COVID-19 exposure. The IL-32 level differences remained comparable across both comparisons (pre- vs. pandemic-era; pre-pandemic vs. diagnosed COVID-19), suggesting that despite elevated IL-32 levels, no differences emerge between asymptomatic exposed subjects and patients with active infections of varying severity.

Secondly, we identified an association between higher circulating IL-32 levels and the first wave of infection, which was associated with late presentation of most severe patients with pneumonia and a high mortality rate. Besides the timing and severity of the disease, it can be speculated that viral strain variations influenced IL-32 expression. It is important to note that throughout the pandemic, the SARS-CoV-2 virus mutated into progressively milder forms, leading to a general reduction in hospitalizations and ICU admissions (37).

Furthermore, we showed that IL-32 levels tended to decrease with increasing age at infection, consistent with the well-established impact of aging on immune function. Age is a major factor influencing COVID-19 severity (38); elderly individuals have a higher risk of severe disease and hospitalization due to comorbidities, whereas younger patients are hospitalized less frequently (39). The observed reduction in IL-32 among older patients may reflect a suppression of proinflammatory responses, potentially due to increased levels of anti-inflammatory cytokines such as IL-10 (40, 41).

Moreover, we observed a strong positive association between IL-32 concentrations and corticosteroid therapy. Corticosteroids, widely used in respiratory diseases for their potent anti-inflammatory effects (42), has been a key clinical intervention for patients with severe COVID-19 (43). Notably, four out of six COVID-19 patients treated with corticosteroids succumbed to the disease after their hospitalization, reflecting a state of particularly severe inflammation. These findings imply that elevated IL-32 levels predominantly reflect the inflammatory response triggered by COVID-19 rather than a direct effect of the viral infection. Supporting this, we found no statistically significant association between IL-32 levels and ICU admission or patients’ survival, consistent with previous studies that did not identify IL-32 as a predictor of disease severity (21). Other recorded comorbidities, including diabetes and cardiovascular diseases, did not show a clear relationship with circulating IL-32 levels.

To better characterize the link of IL-32 with the severity of inflammation, we therefore examined its relationship with two established inflammatory biomarkers implicated in COVID-19 pathophysiology: IL-6 and the NLR. Results revealed no significant association between IL-32 and IL-6; however, IL-32 showed a negative correlation with NLR. Unlike IL-32, NLR and IL-6 have consistently been linked to COVID-19 severity (21), acting as late-stage markers of inflammation after the initial antiviral response. Prior studies reported that distinct IL-32 isoforms may engage in autoregulatory feedback loops depending on the stage and type of infection. For instance, in influenza A infection, the IL-32β isoform downregulates the soluble IL-6 receptor, ultimately leading to a reduction in overall IL-6 levels and an attenuated inflammatory response (44). Therefore, the absence of a direct correlation between IL-6 and IL-32 does not rule out a more intricate, stage-dependent interplay between these cytokines in COVID-19, potentially reflecting a modulatory role of IL-32 in controlling inflammation.

Finally, correlation analysis between IL-32 and endothelial biomarkers in a subset of the clinical cohort did not support a close link between IL-32 and endotheliopathy during severe COVID-19 infection.

A remarkable finding was that we did not observe a significant decline in circulating IL-32 levels in COVID-19 patients who remained in follow-up one year after hospital discharge. In keeping, IL-32 has been reported to be one of the main proteins associated with pulmonary fibrosis, along with IL-8 and IL-10, in post-COVID-19 hospitalized patients, evaluated 6 months after discharge (33).

This observation is in line with a sustained inflammatory response following SARS-CoV-2 infection, with circulating IL-32 remaining implicated during ongoing inflammation. The suggested behavior of IL-32 describes an initial rise in circulating levels alongside the primary antiviral response, followed by a decline as disease progresses, allowing more potent cytokines in late infection stages (more strongly linked to severe symptoms) to take over; however, IL-32 levels never significantly drop below those at infection onset. These data support further investigation of IL-32 as a candidate biomarker for assessing residual inflammation as a long-term consequence of COVID-19. However, we acknowledge the possibility that patients who continued to experience symptoms after infection may have been more likely to stay in follow-up, which could influence these observations.

Limitations of the present study also include the relatively limited sample size, particularly for follow-up evaluations, constraining the power to identify additional significant associations with clinical outcomes and hindering further stratification for confounding factors. Our results started to enlighten the possible involvement of IL-32 in long-COVID or COVID-19 inflammation sequelae, but major limitations remain due to the evolving nature of long-COVID research. Recent studies have begun to clearly define criteria to better characterize the long-COVID phenotype (35). Data recollection and integration will be the starting point to focus our future investigations on IL-32 in the long-COVID context, with clinical data collected more consistently and with less heterogeneity and confounding biases. Additionally, the inability to differentiate among IL-32 isoforms by currently available assays prevented a detailed understanding of post-transcriptional regulation of IL-32 following COVID-19 infection and which isoforms have specific roles in modulating the subsequent inflammatory response. Addressing these limitations will be essential for advancing the characterization of IL-32 expression pathways and for evaluating its potential as a biomarker in future studies, by complementing our cohort observations with functional experiments that establish a mechanistic model.

In conclusion, we found that IL-32 levels increased in apparently healthy individuals concomitantly with a high rate of seroconversion during the first COVID wave, and in patients with severe COVID-19. Among patients, IL-32 was higher in those who were younger and with hyper-inflammation treated with steroids during the first wave. Remarkably, in patients presenting at follow-up with persistent symptoms, IL-32 remained stably elevated.

## Data Availability

The raw data supporting the conclusions of this article will be made available by the authors, without undue reservation.
