# Supplementary material for: Association of SARS-CoV-2 infection with long-lasting increase in circulating IL-32 levels
Source: Front Immunol. 2026 Feb 6;17:1739258. doi: 10.3389/fimmu.2026.1739258 (PMC12920507; doi:10.3389/fimmu.2026.1739258)
Supplement: Supplementary file 1 [file Table1.docx]

Supplementary Material

# Supplementary Figures and Tables

|  | **FIRST WAVE** | **SECOND-FIFTH WAVES** | **p** | **Overall** |
| --- | --- | --- | --- | --- |
| **n** | 58 | 38 |  | 96 |
| **Sex, M** | 40 (69.0) | 28 (73.3) | 0.789 | 68 (70.8) |
| **Age, years** | 57.2 (10.9) | 57.5 (13.3) | 0.907 | 57.3 (11.9) |
| **ICU, yes** | 9 (15.5) | 11(28.9) | 0.184 | 20 (20.8) |
| **Corticosteroid treatment, yes** | 0 (0.00) | 1 (2.6) | 0.830 | 1 (1.0) |
| **Hypertension, yes** | 27 (77.1) | 14 (58.3) | 0.210 | 41 (42.7) |
| **Anti-hypertensive therapy, yes** | 24 (57.1) | 16 (59.3) | 1.000 | 40 (41.7) |
| **Cardiovascular diseases, yes** | 2 (5.7) | 5 (20.8) | 0.176 | 7 (7.3) |
| **Type 2 diabetes, yes** | 5 (14.3) | 4 (16.7) | 1.000 | 9 (9.4) |

Supplementary Table S1 - Hospitalized COVID Patients with Follow-Up. Summary statistics of a subset of hospitalized COVID-19 patients who were screened in follow-up observations. This subcohort was stratified according to the wave of patients’ period of hospitalization (FIRST WAVE/ SECOND-FIFTH WAVES), considering the following variables: Sex, Age, the severity of the disease that led to Intensive Care Unit access (ICU). Clinical features recorded from subjects: corticosteroid treatment, arterial hypertension, antihypertensive treatment, cardiovascular diseases, and type 2 diabetes. p=p-value.

|  | **IL-6** | **NLR** | **Endothelial Biomarkers** |
| --- | --- | --- | --- |
| **n** | 84 | 203 | 42 |
| **Sex, M** | 51 (60.7) | 132 (65.0) | 28 (66.7) |
| **Age, years** | 66.8 (13.0) | 66.6 (14.9) | 61.1 (16.9) |
| **ICU, yes** | 7 (8.3) | 20 (9.9) | 10 (23.8) |
| **Corticosteroid treatment, yes** | 5 (6.0) | 6 (3.0) | 1 (2.4) |
| **Hypertension, yes** | 52 (75.4) | 127 (79.4) | 16 (59.3) |
| **Anti-hypertensive therapy, yes** | 42 (62.7) | 104 (64.6) | 18 (58.1) |
| **Cardiovascular diseases, yes** | 13 (19.4) | 41 (26.5) | 5 (18.5) |
| **Type 2 diabetes, yes** | 14 (20.9) | 38 (24.5) | 7 (25.9) |
| **Wave, first** | 58 (69.0) | 122 (60.1) | 2 (4.8) |
| **Outcome, deceased** | 32 (38.1) | 71 (35.0) | 29 (70.7) |

Supplementary Table S2 - Hospitalized COVID Patients with tested for Pro-inflammatory and Endothelial Biomarkers. Summary statistics of a subset of hospitalized COVID-19 patients who were screened for IL-6 and NLR as pro-inflammatory biomarkers, and thrombomodulin (TM), vascular endothelial growth factor (VEGF), vascular cell adhesion molecule-1 (VCAM-1), and endoglin as endothelial biomarkers. This subcohort was characterized by the following variables: Sex, Age, the severity of the disease that led to ICU admission, and if patients were hospitalized during the period between March 2020 to September 2020 or later (Wave), and survivability. Clinical features recorded from subjects: corticosteroid treatment, arterial hypertension, antihypertensive treatment, cardiovascular diseases, and type 2 diabetes.
